# Different phenotypes of severe flares in patients with systemic lupus erythematosus: results of a clustering analysis in a monocentric cohort

**DOI:** 10.3389/fimmu.2025.1673350

**Published:** 2025-11-21

**Authors:** E. Elefante, D. Schilirò, M. L. Manca, C. Stagnaro, D. Zucchi, C. Cardelli, V. Signorini, M. Maffi, G. Cascarano, R. Zas, L. Carli, F. Ferro, C. Tani, M. Mosca

**Affiliations:** 1 Rheumatology Unit, University of Pisa, Pisa, Italy; 2 Department of Clinical and Experimental Medicine and Department of Mathematics, University of Pisa, Pisa, Italy; 3 Rheumatology Department, Instituto de Investigación Hospital 12 de Octubre, Madrid, Spain

**Keywords:** systemic lupus - erythematosus, lupus flares, cluster analysis, disease outcomes, lupus phenotypes

## Abstract

**Objectives:**

To describe different clinical phenotypes of severe flares in a monocentric cohort of SLE patients and to compare treatment and outcomes.

**Material and methods:**

Retrospective study of prospectively collected data on 122 severe flares occurred in 110 patients, between 2018 and 2023, and followed up for 12 months after the flare. Baseline characteristics included disease activity assessment by SELENA-SLEDAI and BILAG 2004 scores, demographic and laboratory data. A hierarchical unsupervised segmentation method was applied to cluster flares based on baseline features. Treatments and outcomes according to LLDAS, DORIS Remission and SRI definitions, were compared among clusters at different timepoints.

**Results:**

We identified 3 clusters, 2 composed mainly by extra-renal, and one by renal flares. Among non-renal clusters, cluster 1 was characterized by severe constitutional symptoms, serositis and arthritis occurring in younger patients, associated with *hyper-inflammatory* biomarkers and multiple autoantibodies specificities. Cluster 2 included flares with more BILAG B scores and mainly mucocutaneous and musculoskeletal manifestations, and overlapping antiphospholipid syndrome (APS). Cluster 3 was the renal flares cluster. Cluster 1 and the renal cluster were treated more frequently with glucocorticoid (GC) pulses and mycophenolate mofetil (MMF) and presented higher daily and cumulative GCs doses at 12 months (t12). These two clusters also shared similar percentage of attainment of LLDAS (about 50%) and remission (about 35% both) at t12, compared to 73% of LLDAS and 53% of remission in cluster 2 at t12.

**Conclusions:**

We described three different clusters of severe flares in SLE in a real-life setting, identifying a hyper-inflammatory flare phenotype, that shares a comparable proportion of unsatisfying response to treatment as renal flares. Our results may represent a clinical starting point, in the context of precision medicine, for better characterization of severe non-renal disease of SLE, with the final aim of setting up early tailored treatment strategies.

## Introduction

Systemic Lupus Erythematosus (SLE) is characterized by a relapsing-remitting course, alternating quiescent phases and relapses, and by a complex and proteiform clinical picture. Disease flares may be characterized by mild and limited manifestations or present severe and multi-organ forms, up to very aggressive clinical events with organ- and life-threatening manifestations (1). Classically, renal and neuropsychiatric involvement are considered “major” organ involvements and the most severe disease manifestations, with a negative impact on disease outcomes and prognosis (2, 3).

With the availability of new therapeutic options and the implementation of a treat-to-target strategy in the management of SLE, a better definition of different phenotypes of disease flares might support a more personalized and tailored treatment. This is even more important with the advent of new biological drugs for SLE and the possibility of a combined treatment strategy when poor prognostic factors are identified.

In this context, in the latest EULAR recommendations for the management of SLE, it was proposed that early combination therapy, for example with belimumab on top of standard immunosuppressive drugs (IS), ‘should be considered’ in all adult patients with active lupus nephritis, that is by default a severe disease (4). However, it is more difficult to identify aggressive phenotypes and poor prognostic factors for more heterogeneous extra-renal disease.

We have previously demonstrated in our cohort, among extra-renal flares in an outpatient setting, that mucocutaneous flares still represent an unmet need in the management of SLE patients (5).

The aim of the present study was to identify different phenotypes of severe flares, defined according to The Safety of Estrogens in Lupus Erythematosus National Assessment (SELENA)-SLEDAI flare index (SFI) (6), among hospitalized SLE patients regularly followed at our Unit.

## Materials and methods

### Study population

This is a retrospective study of prospectively collected data from a monocentric cohort of adult SLE patients, in regular follow-up at the Lupus Clinic of the University of Pisa, Italy.

All patients enrolled in the study fulfilled the following inclusion criteria:

- patients who met the 2012 SLICC (7) or 2019 EULAR-ACR classification criteria for SLE (8);
- patients who were hospitalized between 2018 and 2023 at our Unit, due to a severe disease flare, according to the SELENA-SLEDAI flare index (SFI) definition (6);
- patients with at least 12 months of regular follow-up available after the SLE flare.

Patients with concomitant infections, oncologic and onco-hematologic diseases were excluded.

For each patient demographic data (sex, ethnicity, age at hospital admission), comorbidities, disease duration, cumulative SLE manifestations and treatments, overlap with Sjogren’s syndrome (SSj) (according to the 2016 ACR/EULAR classification criteria) (9) and antiphospholipid syndrome (APS) (according to the revised Sapporo classification criteria) (10), were retrieved from inpatients’ clinical charts.

The moment of hospitalization due to disease flare was considered as the baseline (t0).

At t0, for each flare the following data were collected: clinical manifestations, immunologic profile, laboratory, imaging and histopathology (if performed during hospitalization), ongoing treatment at admission and therapeutic management of the flare. Particularly, among laboratory routine tests, complete blood cells count, complement factors C3 and C4, immunoglobulins percentage on total serum proteins and IgA, IgG, IgM classes values, inflammatory biomarkers as C reactive protein (CRP), erythrocyte sedimentation rate (ESR), fibrinogen and ferritin were reported.

Lymphadenopathies were assessed by ultrasound.

Biopsy-proven lupus nephritis, when present, was classified according to the International Society of Nephrology/Renal Pathology Society (ISN/RPS) classification (11).

For each flare, disease activity was assessed according to the SELENA-SLEDAI (12) and British Isles Lupus Assessment Group 2004 (BILAG 2004) (13).

After each flare included in the study, we collected data for one year of follow-up. The following time points for disease outcomes and treatment evaluation were set at 3-, 6-, and 12-months of follow-up (reported as t3, t6, and t12).

At each timepoint, disease activity status was assessed according to the Lupus Low Disease Activity State (LLDAS) (14) and Definition Of Remission In SLE (DORIS) (15), and response to treatment was evaluated using the SLE responder index (SRI) criteria (16).

With respect to the therapeutic management, ongoing treatments and therapeutic variations, if any, were collected at t3, t6, and t12. As far as the glucocorticoid (GC) treatment is concerned, we recorded at baseline the GC pulses, if performed, the daily and the cumulative doses of oral prednisone (PDN) administered to treat the flare, and taken by the patient at 1-, 3-, 6- and 12-months’ time-points.

Organ damage was expressed by the Systemic Lupus International Collaborating Clinics Damage Index (SLICC-DI) (17) and assessed at baseline and at t12.

### Statistical analysis

For descriptive analysis, patients and flares characteristics were reported in terms of frequency and percentages for categorical variables and mean and standard deviation or median and interquartile range (based on whether the probability distribution is normal or not) for continuous variables.

#### Clustering analysis methodology

A k-means clustering analysis was performed on the SLE flares selected according to the above inclusion criteria. The analysis utilized Euclidean distance as the similarity metric and included all baseline clinical, laboratory, and immunological features. To determine the optimal number of clusters, we tested solutions with 2, 3, and 4 clusters and compared their performance using silhouette score analysis. The 3-cluster solution was selected based on the highest silhouette score and clinical interpretability of the resulting clusters. No imputation was performed for missing data; complete case analysis was conducted for variables with missing values.

#### Post-hoc analysis

The following *post-hoc* elaborations, 1-way analysis of variance with Bonferroni test for quantitative variables, and Chi square test for qualitative variables, were performed to estimate any statistically significant differences between the clusters.

P-values <0.05 were considered statistically significant.

Data processing was performed with the STATA software package and R statistical software.

This study was conducted according to the Declaration of Helsinki and was approved by the Ethics Committee “Area Vasta Nord Ovest” (CEAVNO) with the number 14478.

## Results

122 severe SLE flares occurring in 110 consecutive patients in the period between 2018 and 2023, fulfilled the inclusion criteria and were considered for the analysis. Overall flares’ characteristics at baseline are summarized in the **Supplementary Table** and briefly reported below.

### Baseline patients’ and flares’ characteristics

Most flares occurred in female patients (83%) of White ethnicity (89%). The mean age and disease duration at the moment of the flare were 39.2 ± 12.3 years, and 8.2 ± 8.6 years, respectively. 22 flares (18%) represented the onset of the disease. The median SELENA-SLEDAI at baseline was 10.5 (IQR 11-16) and more than half of flares (65%) were characterized by “BILAG A” disease manifestations. According to the BILAG definitions, the most frequent active disease manifestations at baseline were renal (46%), followed by musculoskeletal (33%), constitutional (32%) and hematologic (22%). At the moment of hospitalization, 60 cases (49%) were already under treatment with immunosuppressants (IS); 94 cases (77%) were on hydroxychloroquine (HCQ) alone or in combination with IS, and 31 (25%) were not on GC treatment.

### Management, follow-up and outcomes

Glucocorticoids were used in the management of most flares (78%) with pulses administered in 66 cases (54%); 33 flares were treated without introducing (25/33) or modifying the dosage of GCs (8/33), but only introducing an immunosuppressant (the most frequently used were: Mycophenolate Mofetil, Methotrexate, Azathioprine, Rituximab, without a clear predominance of one drug over another). Of note, 4 patients in this group were treated with intravenous immunoglobulins, underlining that they were frail patients at an elevated infectious risk. Compared to GCs-treated flares, the “no-GCs” flares presented more active cutaneous involvement (39% vs 15%; p=0.00) and less frequent renal (24% vs 56%; p=0.002) and cardiopulmonary (p=0.05) active manifestations, significantly lower SLEDAI scores (10.59 ± 6.78 vs 13.9 ± 7.16, p=0.05), and less BILAG A scores in active domains (80% vs 20%; p=0.001).

In 104 flares (85%), ongoing therapy was modified by adding or changing one or more IS. Overall, remission was achieved in almost one third of the cases at 6 and 12 months (26% and 34%, respectively) and LLDAS in almost half of the cases (45% at 6 months and 50% at 12 months).

### Cluster analysis

All 122 flares were considered for the clusterization process. 3 clusters were identified, composed of 40, 34 and 48 flares respectively. Only 10 patients had more than 1 flare; for each of them repeated flares belonged to the same cluster. Cluster 3 included patients with a renal flare, while cluster 1 and 2 were characterized by extra-renal manifestations.

In **Table 1** we summarized the different clinical characteristics, SELENA-SLEDAI and BILAG scores of the three clusters.

**Table 1:** Clusters’ baseline and laboratory features.

Clinical and laboratory variables	Cluster 1 hyperinflammatory (n= 40)	Cluster 2 cold (n= 34)	Cluster 3 renal (n= 48)	P value
Mean age at flare (years, mean ± sd)	38.2 ± 12	46.3 ± 13.9	43.2 ± 11.1	0.007 ^*^
Male sex (%)	15%	14%	16%	ns
Disease duration (years, mean ± sd)	9.1 ± 6	17.5 ± 11.5	15 ± 8.7	0.0001 ^*#^
SELENA SLEDAI t0 (mean ± sd)	13.4 ± 6.1	7.2 ± 4.3	15.9 ± 7.1	0.0001 ^*^^
C3 t0 (mg/dl, mean ± sd)	60.8 ± 20.7	76.3 ± 29	55.9 ± 21.1	0.0007 ^*^^
C4 t0 (mg/dl, mean ± sd)	7.9 ± 6.4	12.3 ± 10.5	10.4 ± 7.3	0.06 ^*^
CRP t0 (mg/dl, mean± sd)	6.4 ± 8	2.9 ± 6.6	0.8 ± 1.1	0.0001 ^*#^
Lymphocyte (t0) (Ux10^9^/L, mean ± sd)	901.2 ± 590.4	1254.2 ± 559.3	1219.7 ± 635.5	0.019 ^*#^
Ferritin (t0) (mg/dl, mean ± sd)	515.4 ± 1107.9	236.3 ± 398.7	190.6 ± 190.7	ns
BILAG A constitutional (%)	57.5%	29%	6%	0.0001
BILAG A cardio-pulmonary (%)	40%	12%	0%	0.0001
BILAG A musculoskeletal (%)	60%	38%	8%	0.0001
BILAG A renal (%)	17.5%	9%	96%	0.0001
Kidney biopsy (%)	5%	9%	54%	0.0001
Arthritis (%)	80%	50%	27%	0.0001
Rash (%)	47.5%	32%	21%	0.032
Fever (%)	70%	18%	6%	0.0001
Lymphadenopathies (%)	82.5%	33%	36%	0.0001
Proteinuria/casts (%)	20%	9%	96%	0.0001
Cumulative IS (n; mean ± sd)	3.6 ± 1.5	3 ± 1.8	4.1 ± 1.9	0.019 ^^^
Anti-Sm (%)	50%	6%	27%	0.0001
Anti-nucleosome (%)	80%	21%	35%	0.0001
Anti-hystone (%)	67.5%	18%	31%	0.0001
Anti-dsDNA (%)	95%	45%	83%	0.0001
APS (%)	5%	26%	15%	0.0001

Statistical significance * between 1 and 2, # between 1 and 3, ^ between 2 and 3.

CRP, C-reactive protein; APS, Antiphospholipid antibody syndrome; ns, not statistically significant; t0: baseline.

### Clinical and laboratory features of the three clusters at baseline

Cluster 1 included flares that occurred in younger patients (mean age 38.2 ± 12 years), with shorter disease duration (9.1 ± 6 years). Overall, arthritis (80% vs 50% and 27%), fever (70% vs 18% and 6%) and lymphadenopathies (82.5% vs 33% and 36%) resulted significantly more frequent in this cluster compared to the others. Constitutional involvement appeared significantly associated with this cluster, also characterizing the past medical history of patients (74% vs 47% and 37% cluster 2 and 3 respectively; p= 0.003). No significant differences in other cumulative disease manifestations were found between clusters. 2 kidney biopsies were performed in Cluster 1, showing one class II and one class IV lupus nephritis.

As far as laboratory parameters is concerned, cluster 1 included flares characterized by marked CRP elevation (6.4 ± 8 vs 2.9 ± 6.6 and 0.8 ± 1.1 mg/dL, p=0.000), a trend for higher ferritin serum levels and lymphopenia (901.2 ± 590.4 vs 1254.2 ± 559.3 and 1219.7 ± 635.5 Ux10^9^/L, p=0.000) compared to the other clusters. Cluster 1 was also characterized by a higher number of different SLE-specific autoantibodies (anti-histone, anti-Sm, anti-nucleosome and anti-dsDNA antibodies) than cluster 2 and 3 (p=0.000). Interestingly, despite a shorter disease duration, this cluster presented a number of cumulative previous IS comparable to cluster 3.

Considering the most prevalent clinical and laboratory features of flares belonging to cluster 1, we named it the “hyper-inflammatory” cluster.

Cluster 2 included flares presenting lower disease activity (**Table 1**), and less severe clinical manifestations, mainly in the mucocutaneous and musculoskeletal domains, reporting more “BILAG B” scores compared to the other clusters (59% vs 28% and 19%; p=0.000). In three cases, a kidney biopsy was required, showing one class III, one class II and one class IV lupus nephritis.

In line with a “milder” clinical profile, flares in cluster 2 presented higher values of complement C3 (76.3 ± 29 vs 60.8 ± 20.7 and 55.9 ± 21.1 mg/dL; p=0.000). Patients belonging to this cluster were older, with longer disease duration with respect to cluster 1 (p=0.000), requiring fewer cumulative IS in their history with respect to cluster 3 (3 ± 1.8 vs 4.1 ± 1.9, p=0.019). Moreover, a significant higher percentage of patients in this cluster presented a secondary APS (26% vs 5% and 15%; p=0.000) compared to cluster 1 and 3. However, no significant differences emerged between the clusters regarding the positivity of the different antiphospholipid antibodies.

We named cluster 2 the “cold” cluster to suggest a lower severity of flares and a different clinical and laboratory phenotype in contrast to that of cluster 1.

Cluster 3 included most flares with active renal involvement presenting a BILAG score A in the renal domains (96% vs 17.5% and 9%, p= .0001). As expected, more than a half of cases in this cluster (26/48 flares) underwent a renal biopsy, and histologic examination showed a proliferative glomerulonephritis in most of the cases: 18/26 (70%) were class III or IV; in 4/26 (15%) cases, class III or IV was in combination with class V.

As far as serology is concerned, a clear prevalence of anti-dsDNA positivity compared to other autoantibodies was observed.

Cluster 3 was named the “renal” cluster.

### Therapeutic management of flares in the three clusters

The therapeutic management of the three clusters at t0 is summarized in **Table 2**.

**Table 2:** Therapeutic management in the three clusters.

Clinical and laboratory variables	Cluster 1 hyperinflammatory (n= 40)	Cluster 2 cold (n= 34)	Cluster 3 renal (n= 48)	p value
GCs pulses (%)	50%	15%	83%	0.0001
Add/change IS t0 (%)	85%	85%	86%	ns
MMF t0 (%)	30%	9%	47%	0.001
CTX t0 (%)	10%	6%	23%	ns
RTX t0 (%)	7.5%	9%	19%	ns
Belimumab t0 (%)	25%	30%	8.5%	0.025
AZA t0 (%)	7.5%	9%	4%	ns
MTX t0 (%)	10%	15%	4%	ns
CyA t0 (%)	2.5%	3%	2%	ns
Cum GCs dose t1 (mg ± sd)	1059.7 ± 722.2	581.3 ± 621.8	1590.9 ± 912.4	0.027# 0.0001^ 0.027*
GCs t3 (mg/daily)	7.9 ± 7.2	5.8 ± 7.1	9.9 ± 8	ns
Cum GCs dose t3 (mg ± sd)	1647.1 ± 1093.1	941.4 ± 816.1	2058.9 ± 1160.0	0.017* 0.0001^
GCs t6 (mg/daily)	5.8 ± 4.6	4.6 ± 6.4	7.5 ± 9	ns
Cum GCs dose t6 (mg ± sd)	2268.3 ± 1323.	1314.0 ± 994.6	2738.8 ± 1648.0	0.018* 0.0001^
GC t12 (mg/daily)	6.3 ± 6.2	3.5 ± 2.2	5 ± 4.7	0.049*
Cum GCs dose t12 (mg ± sd)	3081.5 ± 1410.4	1865.7 ± 1230.2	3670.7 ± 1884.9	0.007* 0.0001^

Statistical significance * between 1 and 2, # between 1 and 3, ^ between 2 and 3.

t0: baseline; t3, t6, t12: timepoints at 3-6–12 months after the flare. GCs, Glucocorticoids; IS, Immunosuppressant; MMF, Mycophenolate Mofetil; CTX, Cyclophosphamide; RTX, Rituximab; AZA, Azathioprine; MTX, Methotrexate; CyA, Cyclosporine; ns, not statistically significant.

A significant difference was observed in the administration of GC pulses which were more commonly used to treat flares in clusters 1 and 3. No differences emerged between clusters as for the number of flares treated without GC (data not shown). About 85% of flares in each cluster required a modification of therapy by adding or changing an IS. MMF was the most used drug in clusters 1 and 3.

Belimumab was added in 25% and 30% of flares in clusters 1 and 2 reflecting the fact that most flares included in our study occurred before the approval of BLM for lupus nephritis. No significant differences emerged with respect to other therapies.

At t12 cluster 1 and cluster 3 had been treated with significantly higher GC cumulative doses compared to cluster 2. Interestingly, cluster 1 presented the highest daily GC dose at t12 (6.3 ± 6 mg/daily vs 3.5 ± 2 and 5 ± 4.7 in clusters 2 and *3*, respectively) (**Table 2**).

### Disease outcomes in the three clusters

We finally compared the attainment of treatment targets after flare in the three clusters.

In the cluster 2 an early achievement of LLDAS and remission at t3 was observed in a statistically significant higher percentage of cases compared to the clusters 1 and 3 (p= 0.0001 and 0.0014, respectively). Flares in the clusters 1 and 3 showed a similar course during follow-up, presenting a comparable and quite low percentage of patients that achieved LLDAS and remission at the different timepoints. Particularly, only about one third of flares reached remission at t12 in both clusters 1 and 3 (35%) (**Table 3**).

**Table 3:** Outcomes in the three clusters.

Clinical and laboratory variables	Cluster 1 hyperinflammatory (n= 40)	Cluster 2 cold (n= 34)	Cluster 3 renal (n= 48)	p value
SLEDAI t3 (mean ± sd)	4.9 ± 3.6	3. ± 3.2	9.0 ± 5.3	0.0001 ^#^^
LLDAS t3 (%)	29%	68%	9%	0.0001
Remission t3 (%)	13%	32%	9%	0.014
SLEDAI t6 (mean ± sd)	4.3 ± 3.8	3.0 ± 2.5	7.0 ± 5.5	0.0001 ^#^^
LLDAS t6 (%)	49%	66%	33%	0.016
Remission t6 (%)	29%	37%	20%	ns
Non responders t6 (%)	27%	16%	38%	ns
New *flare* t6 (%)	11%	9%	4%	ns
SLEDAI t12 (mean ± sd)	4.7 ± 3.5	2.5 ± 2.2	6.1 ± 6.3	0.003 ^#^^
LLDAS t12 (%)	51%	73%	47%	ns
Remission t12 (%)	35%	53%	35%	ns
Non responders t12 (%)	16%	10%	30%	ns
New *flare* t12 (%)	21%	11%	9%	ns
SLICC-DI t12 (mean ± sd)	0.8 ± 1	1.4 ± 1.9	1.3 ± 1.7	ns

Statistical significance * between 1 and 2, # between 1 and 3, ^ between 2 and 3.

t3, t6, t12: timepoint at 3-6–12 months after the flare. LLDAS, Lupus Low Disease Activity State; ns, not statistically significant.

No significant differences were observed between clusters in the percentage of non-responders and new disease flares at t6 and t12.

Finally, no significant differences between clusters were found as far as damage accrual after one year of follow-up is concerned. In particular, 76 flares (62%) had no damage at t0. Among them, 15 (20%) developed damage at t12, 4 belonging to cluster 1, 2 to cluster 2 and 9 to cluster 3 (p=0.2). Moreover, at baseline 48 flares (40%) already presented a SLICC-DI score ≥ 1, with a mean value of 1.95 ± 1, and at t12 this percentage rose to 50% of flares with a mean SLICC-DI value of 2.03 ± 1.5, with no significant differences between clusters. Overall, at t12, 27 flares presented a damage accrual, without differences between clusters.

## Discussion

The main objective of this study was to identify different phenotypes of severe flares in a monocentric cohort of SLE patients. Among 122 consecutive SLE flares requiring hospitalization at our Unit between 2018 and 2023, we identified three distinct clinical and biological clusters of severe flares: one cluster constituted mainly by *“renal flares”* (cluster 3), and two by extra-renal flares: cluster 1, characterized by “*hyperinflammatory flares”*, and cluster 2, by milder and “*cold flares”*.

In the current literature, several studies have tried to identify different subsets of SLE patients, underlining the complexity of SLE and the need for physicians to stratify the therapeutic strategies according to the different patients’ characteristics. Pisetzky and colleagues have recently introduced the concept of two different types of SLE: Type 1 SLE characterized by the classical inflammatory symptoms of the disease, and Type 2 SLE characterized by symptoms such as fatigue, widespread body pain, neuropsychiatric symptoms, and sleep disturbance that are often unrelated to disease activity (18).

Overall, starting from the immunological profile or from the cumulative clinical features of the disease in large US, European and Asian cohorts, currently available data seem to converge on the existence of some subsets of patients. Particularly, patients with dominant proliferative nephritis present anti-dsDNA positivity and a higher risk of damage accrual, while patients with anti-RNP positivity seem to have lower probability of renal involvement and an association with extra-renal manifestations, like myositis and anemia. Patients with associated Sjogren’s syndrome are mainly female and seem to have milder clinical manifestations of SLE, while patients with anti-phospholipid antibodies are clearly at higher risk of vascular complications, neurological involvement and damage accrual (19–21).

Recently, in a very large multicenter cohort of SLE patients in China, three different subgroups were identified among “serologically active clinically quiescent (SACQ)” patients: cluster 1 consisted of elderly males with a history of major organ involvement and the highest risk of severe flares and damage accrual; cluster 2 was characterized by milder disease and a lower risk of organ damage, with 30.8% of patients in this cluster who successfully discontinued low-dose glucocorticoids; cluster 3 presented the highest proportion of lupus nephritis and a moderate risk of organ damage (22).

To the best of our knowledge, our study is the first one performing a clustering-analysis focusing on clinical, laboratory and immunologic features of severe SLE flares, defined according to the SFI criteria. The clustering analysis, performed on flares instead of cumulative clinical features, proved to be a useful approach to understanding the heterogeneity of severe SLE flares. By moving beyond traditional clinical classifications, we were able to reveal distinct phenotypes that share underlying biological and clinical characteristics. This method allowed for a more nuanced characterization of disease severity, potentially paving the way for more targeted therapeutic strategies.

As expected, and in line with literature data (21, 23, 24), one of the identified clusters included flares with prevalent renal expression, thus characterized by active lupus nephritis associated with a prevalent positivity of anti-dsDNA antibodies. More interestingly, two other distinct clusters of non-renal severe flares emerged in our study.

Cluster 1, which we called the “*hyperinflammatory”* cluster, included younger patients with a shorter disease duration and was characterized, from a clinical point of view, by constitutional manifestations (such as fever and lymphadenopathies), severe arthritis and cardiopulmonary manifestations. Moreover, from a biological point of view, it was characterized by hyperinflammatory response with marked elevation of CRP and ferritin levels, lymphopenia, and multiple SLE specific autoantibodies positivity.

Historically, high CRP levels in SLE are considered more suggestive of infection rather than disease activity (25), although a moderate elevation of CRP has been associated with specific SLE manifestations, namely arthritis and serositis (26, 27). However, our results outlined a more definite phenotype of SLE flare for which a marked elevation of all the inflammatory biomarkers represents the hallmark of disease activity and is associated with a severe clinical picture and with a quite complex disease history, rather than with a single disease manifestation.

Cluster 2, the other extra-renal cluster, which we called the “*cold”* cluster, was characterized by milder clinical manifestations, mainly mucocutaneous and articular, and by a higher prevalence of APS. Despite this apparent benign presentation, all the flares in this cluster should also be considered severe, as they required hospitalization and/or therapeutic escalation.

As already stated, renal involvement represents the prototype of a severe SLE manifestation and current literature offers a great amount of data on its therapeutic management and treatment targets. On the contrary, less is known with respect to other severe disease phenotypes.

Interestingly, in our study, several similarities emerged between the *hyperinflammatory* and the *renal* clusters in terms of clinical severity, therapeutic choices, and outcomes.

At baseline, these two clusters presented similar total SLEDAI score and complement consumption, and they shared analogous therapeutic attitude in the use of GCs. The *hyperinflammatory* flares required high doses of GCs, with pulses needed in half of the cases. During the follow-up, no differences emerged in terms of cumulative GC doses, at the different timepoints, between *hyperinflammatory* and *renal* flares. Moreover, MMF was the most frequently used IS to treat flares in both clusters. Interestingly, BLM was the second drug administered in the *hyperinflammatory* cluster (25%), suggesting that biological drugs could represent a valuable option for this disease phenotype. In the renal subgroup, BLM was less used as most of the flares included in the study occurred before its approval for lupus nephritis.

As for the response to treatment at the different timepoints during the follow-up, the *hyperinflammatory* and the *renal* clusters presented a comparable and quite low percentage of flares that achieved the targets of LLDAS and remission; in particular, at t12, only about one-third of flares in both clusters had reached remission. Of course, our study presents some limitations. First, the limited follow-up period of 12 months compromised the possibility to draw conclusions on longer-term outcomes in terms of response to treatment and, above all, damage accrual in the different clusters. Another limitation is that most of the flares included in the study occurred before the approval of belimumab for lupus nephritis and all of them occurred before the approval of anifrolumab for non-renal SLE. So, maybe the therapeutic approach to flares included in the study, nowadays, could have been changed according to the latest EULAR recommendations for the management of SLE.

It is interesting to underline that some of the clinical and laboratory features that we described for the *“hyperinflammatory”* flares, have been associated, according to recent literature data, with a high interferon (IFN) signature, particularly fever, lymphopenia, hypocomplementemia, multiple autoantibodies positivity and resistance to glucocorticoids (28–30). So, we could only speculate whether the *“hyperinflammatory”* flares identify a subset of IFN-driven SLE flares that may benefit from an early introduction of targeted anti-IFN treatment.

Finally, the relatively small size of each cluster did not allow us to perform subanalysis to evaluate the response to different treatments within each flare phenotype.

We also believe that our study presents some points of strength. First, a large amount of data for each flare, with few missing, was collected in a homogeneous and prospective way despite the retrospective design of the study. Moreover, we believe that the monocentric design of the study ensures a homogeneous approach in the management of these flares. In conclusion, this study describes three different phenotypes of severe flares in SLE in a real-life setting. In particular, we identified a kind of non-renal flare presenting with fever, arthritis and serositis associated with a *hyperinflammatory* response. These flares appeared to be as severe as the “classical” severe renal flares, deserving similar aggressive therapeutic strategies and burdened by a comparable proportion of unsatisfying response to treatment.

In the era of precision medicine, a better phenotyping of SLE patients and flares appears of utmost importance, especially for non-renal manifestations, for the early identification of flares with poor prognostic factors deserving early aggressive treatments. In this context, the advent of biological drugs and early combined treatment strategies could help in the management of severe flares, improving the attainment of treatment targets and disease outcomes.

## Data Availability

The original contributions presented in the study are included in the article/**Supplementary Material**. Further inquiries can be directed to the corresponding author/s.
