# Supplementary material for: Different phenotypes of severe flares in patients with systemic lupus erythematosus: results of a clustering analysis in a monocentric cohort
Source: Front Immunol. 2025 Nov 21;16:1673350. doi: 10.3389/fimmu.2025.1673350 (PMC12678312; doi:10.3389/fimmu.2025.1673350)
Supplement: Supplementary file 1 [file Table1.docx]

Supplementary Table: *Flares’ characteristics*

| **Overall flares (n= 122)** | | | |
| --- | --- | --- | --- |
| Disease duration at t0 (years, mean ± sd) | 8.2 ± 8.6 | BILAG t0 Hematological (%) | 22% |
| SLICC DI t0 (mean ± sd) | 0.77±1.2 | BILAG t0 Cardio-pulmonary (%) | 16% |
| SELENA-SLEDAI t0 (median, IQR) | 10,5 (11-16) | BILAG t0 Musculoskeletal (%) | 33% |
| BILAG t0 Constitutional (%) | 32% | BILAG Mucocutaneous (%) | 19% |
| BILAG t0 Renal (%) | 46% | Constitutional BILAG A (%) | 9% |
| BILAG t0 Neuropsychiatric (%) | 3.2% | Musculoskeletal BILAG A (%) | 21% |
| Cardio-pulmonary BILAG A (%) | 15% | Hematological BILAG A (%) | 6.3% |
| Renal BILAG A (%) | 49% | Immunoglobulins (%, mean ±sd) | 20.3±5.9 |
| C3 (mg/dl, mean ± sd) | 63±24 | Anti-dsDNA (%) | 107/122, 87.7% |
| C4 (mg/dl, mean ± sd) | 10±8 | Anti-C1q (%) | 48% |
| Fibrinogen (mg/dl, mean) | 398±142 | Anti-Ro52 (%) | 55% |
| CRP (mg/dl, mean ± sd) | 3.2±6.2 | Anti-Ro60 (%) | 43% |
| ESR (mm/h, mean ± sd) | 49.6±29.5 | Anti-SSB (%) | 17% |
| Ferritin (mg/dl mean ± sd) | 289±682 | Anti-Sm (%) | 31.8% |
| Hb (g/dl, mean ± sd) | 12±1.6 | Anti-Nucleosome (%) | 51% |
| Platelets (Ux10^9^/L, mean ± sd) | 242±174 | Anti-Hystone (%) | 43.6% |
| Lymphocytes (Ux10^9^/L, mean ± sd) | 1124±615 | Anti-RNP (%) | 50.9% |
| Neutrophils (Ux10^9^/L, mean ± sd) | 3371±1975 | Anti-B2GPI IgM (%) | 7.2% |
| LAC (%) | 24.5% | ACLA IgG (%) | 26% |
| Anti-B2GPI IgG (%) | 11.8% | ACLA IgM (%) | 17% |
| Arthritis* (%) | 56.3% | Pleuritis* (%) | 17.2% |
| Rash* (%) | 36.3 | Pericarditis* (%) | 12% |
| Leukopenia* (%) | 39% | Lymphadenopathies (%) | 56.3% |
| Thrombocytopenia* (%) | 11% | Splenomegaly (%) | 21% |
| Fever* (%) | 33.6% | Length of hospitalisation (days, mean ± sd) | 15±10 |
| Vasculitis* (%) | 2.7% | 6MP/daily t3 (mg/daily, mean ± sd) | 8.2±7.7 |
| Proteinuria* (%) | 52% | LLDAS t3 (%) | 32% |
| GCs pulses (%) | 59% | Remission t3 (%) | 17% |
| Add/change IS or HCQ (%) | 104/122 (85%) | SLEDAI t6 (mean ± sd) | 4.7±4.6 |
| MMF t0 (%) | 32% | 6MP/daily t6 (mg/daily, mean ± sd) | 11.3±34.3 |
| CTX t0 (%) | 15% | LLDAS t6 (%) | 45.4% |
| RTX t0 (%) | 14.5% | Remission t6 (%) | 26% |
| AZA t0 (%) | 7.2% | SLEDAI t12 (mean ± sd) | 4.7±4.8 |
| MTX t0 (%) | 10% | 6MP/daily t12(mg/daily, mean ± sd) | 5±5 |
| BLM t0 (%) | 21% | LLDAS t12 (%) | 50% |
| Colchicine t0 (%) | 11% | Remission t 12 (%) | 34% |
| IVIg t0 (%) | 8% | SLICC DI t12 (mean ± sd) | 1.14 ± 1.5 |
| SLEDAI t3 (mean ± sd) | 6±4 | PDN cumulative dose t6 (mg, mean ± ds) | 2736 ± 1862.5 |
| SLICC DI variation t0-t12 (%) | 14% | PDN cumulative dose t12 (mg, mean ± ds) | 3722 ± 2147.5 |

*According to SELENA-SLEDAI definitions. t0: baseline; t3, t6, t12: timepoints at 3-6-12 months after the flare; CRP: C-reactive protein; ESR: Erythrocyte Sedimentation Rate; Hb: haemoglobin; anti-B2GPI: anti-beta2 glicoprotein I antibodies; LAC: lupus anticoagulant; ACLA: anticardiolipin antibodies; GCs: Glucocorticoids; 6MP: 6-methylprednisolone; IS: Immunosuppressant; HCQ: Hydroxychloroquine; MMF: Mycophenolate Mofetil; CTX: Cyclophosphamide; RTX: Rituximab; AZA: Azathioprine; MTX: Methotrexate; BLM: Belimumab; IVIg: Intravenous Immunoglobulins; PDN: oral prednisone; LLDAS: Lupus Low Disease Activity State.
